# Origin and significance of the human DNase repertoire

**DOI:** 10.1038/s41598-022-14133-w

**Published:** 2022-06-20

**Authors:** Giulia Mori, Danila Delfino, Paola Pibiri, Claudio Rivetti, Riccardo Percudani

**Affiliations:** Department of Chemistry, Life Sciences and Environmental Sustainability, University of Parma, 43124 Parma, Italy

**Keywords:** Computational biology and bioinformatics, Evolution, Genetics

## Abstract

The human genome contains four *DNase1* and two *DNase2* genes. The origin and functional specialization of this repertoire are not fully understood. Here we use genomics and transcriptomics data to infer the evolutionary history of DNases and investigate their biological significance. Both DNase1 and DNase2 families have expanded in vertebrates since ~ 650 million years ago before the divergence of jawless and jawed vertebrates. *DNase1*, *DNase1L1*, and *DNase1L3* co-existed in jawless fish, whereas *DNase1L2* originated in amniotes by tandem duplication of *DNase1*. Among the non-human DNases, *DNase1L4* and newly identified *DNase1L5* derived from early duplications that were lost in terrestrial vertebrates. The ancestral gene of the DNase2 family, *DNase2b*, has been conserved in synteny with the *Uox* gene across 700 million years of animal evolution,while *DNase2* originated in jawless fish. DNase1L1 acquired a GPI-anchor for plasma membrane attachment in bony fishes, and DNase1L3 acquired a C-terminal basic peptide for the degradation of microparticle DNA in jawed vertebrates. The appearance of DNase1L2, with a distinct low pH optimum and skin localization, is among the amniote adaptations to life on land. The expansion of the DNase repertoire in vertebrates meets the diversified demand for DNA debris removal in complex multicellular organisms.

## Introduction

DNases are key enzymes in human health. Their presence throughout the animal kingdom underlines their importance in the development and homeostasis of metazoans. DNases are endonucleases that catalyze the degradation of DNA through the hydrolysis of the phosphodiester bond. Although DNA molecules originate both from endogenous and exogenous sources, the major recognized target of DNase action is self-DNA, which can be found both inside and outside the cell and has to be constantly degraded to maintain homeostatic conditions in the body^1–3^. Indeed, normal tissue development requires the renewal, through programmed cell death, of billions of cells every day, whose nuclear and mitochondrial DNA has to be efficiently eliminated^4^. In particular districts, such as the hematopoietic system, skin, and eye lens, nuclei extruded from differentiating cells are a prominent source of self-DNA that, unless removed, would become an obstacle to tissue and organ function, causing disorders such as anemia, parakeratosis, and cataract^5–7^. Furthermore, the innate DNA sensors do not completely discriminate between foreign and self-DNA, and consequently self-DNA has to be kept below a certain “immunostimulatory” threshold to avoid the aberrant activation of the immune system^8^. Under abnormal circumstances of cell and tissue damage, the enhancement of apoptosis/necrosis and the process of NETosis, whereby dying neutrophils release large quantities of DNA in the form of neutrophil extracellular traps (NETs), result in increased levels of self-DNA in the circulation, that could trigger autoimmunity^9^. Therefore, DNases have evolved as essential safeguard mechanisms that function extra- and intracellularly to contribute to the clearance of self-DNA under physiological conditions and curb the oversupply of self-DNA under pathological conditions, thereby preventing the detrimental effects of its accumulation^10^.

**Figure 1:**
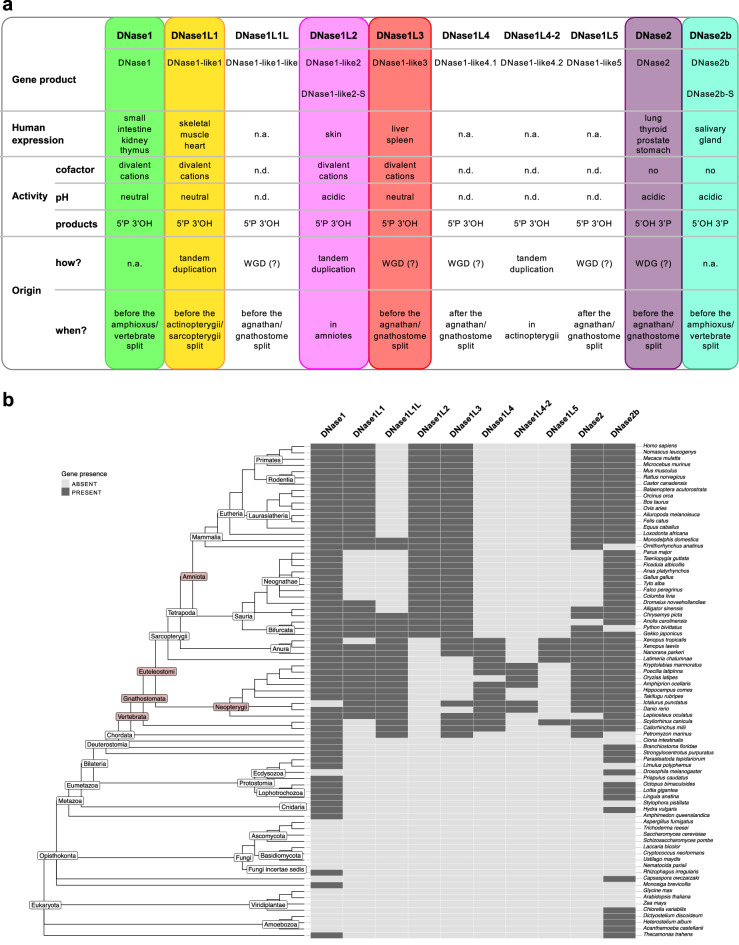
Overview of vertebrate DNase1 and DNase2 and gene distribution in eukaryotes. (**a**) Main features of DNase1 and DNase2 enzymes. Gene expression has been inferred from two human RNA-seq datasets from NCBI (Supplementary Table S1). The mechanism of gene duplication has been tentatively indicated as whole genome duplication (WGD) in the absence of any evidence of tandem duplication. (**b**) Distribution map of presence and absence of *DNase1* and *DNase2* genes as defined by HMM-based search in complete genomes. The tree represents eukaryote phylogeny according to NCBI classification. Taxonomic groups with DNase duplication events are highlighted in color.

Among the enzymes with endonucleolytic activity, the term “DNase” is attributed to two distinct protein families, DNase1 and DNase2, which differ in structural and catalytic properties^11^ (Fig. 1a). DNase1 requires divalent cations for activity, acts mainly at neutral pH, and cleaves DNA producing 5′-P and 3′-OH ends^12–14^. DNase2 does not need any cofactor, has an acidic pH optimum, and generates 5′-OH and 3′-P ends^15,16^.

In humans, the DNase1 family comprises two serum enzymes, DNase1 and DNase1L3, both involved in the degradation of DNA from apoptotic, necrotic, and NETosing cells^17,18^; an ecto-enzyme, DNase1L1, for the digestion of extracellular DNA in skeletal and heart muscle and different types of migrating cells^19^; a tissue specific enzyme, DNase1L2, whose role has been described in skin differentiation^6^. The DNase2 family includes two lysosomal enzymes, DNase2, which is mainly produced by macrophages and processes engulfed DNA from apoptotic cells and erythroid precursors^5^, but it was also shown to exert a crucial function in holocrine secretion of sebum^20^ and DNA degradation at the skin surface^21^, and DNase2b, which is expressed in salivary glands and few other tissues^22^ without a clearly defined function, although its implication during lens fibers differentiation has been demonstrated in mice^7^. Consistent with their fundamental role in the disposal of self-DNA, the absence of DNase1 or DNase2 family members has various clinical consequences, including autoinflammatory and autoimmune diseases. For instance, loss-of-function mutants of DNase1 and DNase1L3 have been associated with Systemic Lupus Erythematosus (SLE)^23,24^, DNase1L2 with psoriasis^25^, and DNase2 with anemia and arthritis^26^. The DNase endonucleolytic activity has been exploited as a therapeutic strategy in the treatment of different types of respiratory diseases, such as cystic fibrosis (CF), asthma, chronic lung diseases, and COVID-19 infection^27,28^. DNase1 is currently used in CF patients as a first-line mucolytic agent^29^, and we recently proposed DNase1L2 as a promising alternative for CF therapy^30^.

While the full physiological significance of the DNase repertoire is not yet understood, it is clear that the multiplicity of DNases is a conserved feature across vertebrates. However, less clear are the origin and evolution of vertebrate DNases, the understanding of which may shed light on the significance of the DNase diversity. First, there is no consensus phylogeny of vertebrate DNases. DNase1 and DNase2 have previously been analyzed from a phylogenetic perspective as single proteins^31,32^. However, not much attention has been paid to the evolutionary history of the two complete DNase families. For instance, it remains unknown which DNases were present in ancestral vertebrates, and which DNases emerged and when during vertebrate evolution. Second, the distribution of DNase genes across vertebrate lineages has not yet been evaluated systematically and comprehensively. There is only sporadic evidence of gene gains and losses in the history of the two families, such as the lack of DNase2 in birds^33^. Third, a unified nomenclature for vertebrate DNases is missing and database annotations are often ambiguous or inaccurate.

In this work, we used genomics and transcriptomics data to infer the evolutionary history of DNase1 and DNase2 families, including DNases that are not present in humans, but are part of the vertebrate repertoire. We provide a phylogenetic reconstruction of the two DNase families, an account of the gene gains and losses, and a revised classification of the vertebrate DNases. This enabled us to elucidate the ancestral gene of each family, and the time and mechanism of the appearance (when and how) of their descendants. A probable scenario for the observed changes in the DNase repertoire during vertebrate evolution is proposed.

## Results

### Vertebrate expansion of the *DNase1* and *DNase2* genes

To trace the origin of the human DNase1 and DNase2 families, we started by determining the occurrence of the different *DNase* genes in eukaryotes. First, we searched for orthologs of the six human *DNase* genes in all the complete genomes of vertebrates available in the Ensembl database. The search was then repeated for orthologs and paralogs of DNase genes from *Gallus gallus* (*Gg*) and *Danio rerio* (*Dr*). The retrieved protein sequences were analyzed for phylogenetic relationships and conserved synteny (see below) to classify the different DNase groups; the four DNase genes without a human ortholog were classified in distinct orthogroups named as: DNase1L1L, DNase1L4, DNase1L4-2, and DNase1L5 (Fig. 1a). For each of the overall ten orthogroups, a representative set of 10–20 protein sequences was used to build a Hidden Markov Model (HMM) that was employed for homology search in eukaryotic proteomes. The data show that *DNase1* and *DNase2* genes are present in a wide range of animals, but are absent in fungi and plants, with the exception of glomeromycetes (*DNase1*) and green algae (*DNase2*) (Fig. 1b), in accordance with a previously published phylogenetic study of DNase2^31^. In addition, *DNase1* or *DNase2* genes are found in few unicellular eukaryotes, such as *M. brevicollis* (Choanoflagellata), *C. owczarzaki* (Filasterea), and *T. trahens* (Apusozoa). We found a minimal DNase repertoire comprising one *DNase1* and one *DNase2* (*DNase2b*) gene in basal Eumetazoa, encompassing invertebrate protostomes such as arthropods (e.g. *P. tepidariorum*) and molluscs (e.g. *O. bimaculatus*), and early deuterostomes such as echinoderms (e.g. *S. purpuratus*) and lancelets (e.g. *B. floridae*) (Fig. 1b). It can be seen that a first major expansion of this DNase repertoire took place in primitive vertebrates (agnathans or jawless fish such as lampreys e.g. *P. marinus*) and involved *DNase1L1L*, *DNase1L3*, and *DNase2* (Fig. 1b). A second expansion took place in higher vertebrates (gnathostomes or jawed vertebrates) and involved *DNase1L4* and *DNase1L5* (Fig. 1b). Three other expansions occurred in euteleostomes (bony vertebrates; *DNase1L1*), in Neopterygii (bony fish; *DNase1L4-2*), and in amniotes (*DNase1L2*) (Fig. 1b). Thus, a spread of *DNase* gene families has occurred in vertebrates, suggesting the requirement for a greater assortment of DNase proteins with respect to non-vertebrate metazoans. The complete vertebrate DNase repertoire includes eight *DNase1* orthogroups, four of which are present in mammals, and two *DNase2* orthogroups that are both present in mammals. Among the newly defined orthogroups, *DNase1L1L* and *DNase1L4* emerged early in vertebrate evolution and were maintained in reptiles and amphibians, but not in mammals; *DNase1L5* also emerged early, but was maintained only in some cartilaginous fishes (e.g. *C. milii* and *S. canicula*), in sarcopterygian fishes (e.g. the coelacanth *L. chalumnae*), and in amphibians; *DNase1L4-2* showed the most restricted distribution, being found only in, but not in all, bony fishes (Fig. 1b).

### Evolutionary and functional relatedness of DNase1 proteins

**Figure 2:**
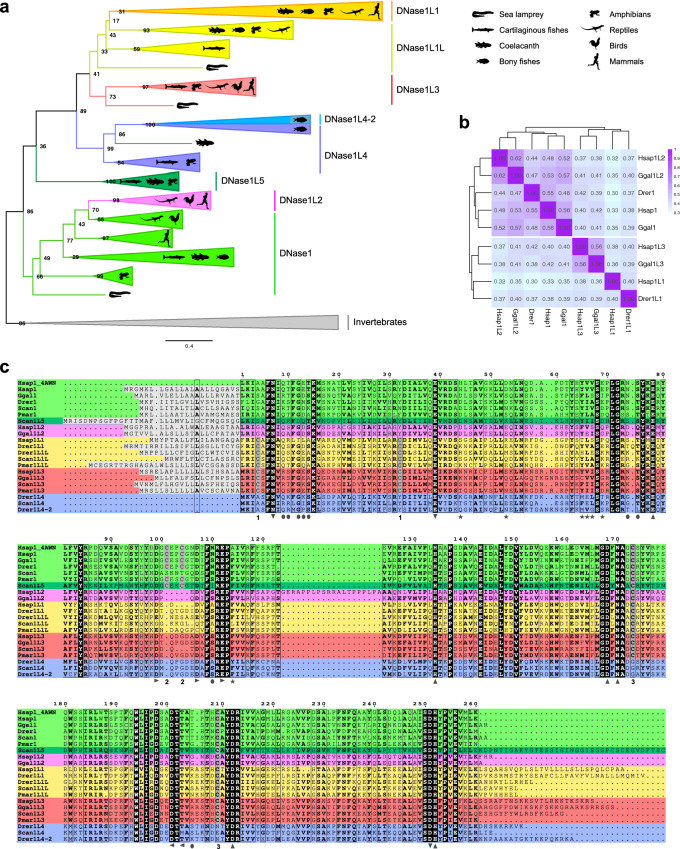
Origin and conservation of DNase1 family in vertebrates. (**a**) Maximum-likelihood phylogeny of 237 DNase1 family proteins inferred with the LG substitution model. Tree branches were collapsed into subgroups representing DNase1 proteins in different groups of organisms. Invertebrate sequences were used to root the tree. Bootstrap values are shown at the internal nodes. Scale bar, substitution/site. The full tree is shown in Supplementary Fig. S1. (**b**) Clustered heatmap of pairwise sequence identity of DNase1 family proteins. Sequences are indicated with the abbreviated taxon name followed by the DNase1 group identifier according to our classification. (**c**) Multiple alignment of DNase1 family sequences from selected species. Remarkable residues are indicated as follows: catalytic, up triangles; Mg^2+^ binding, down triangles; Ca^2+^ binding in site I, right triangles; Ca^2+^ binding in site II, left triangles, actin binding, stars; DNA binding, circles; cysteine pairs forming disulfide bonds, same number and dark grey highlight. The signal peptides predicted with SignalP 5.0 are highlighted in light grey. The DNase1L5 sequence has been truncated at the C terminus; the complete sequence is shown in Supplementary Fig. S3.

To understand the origin and the evolutionary relatedness of the DNase1 proteins, phylogenetic reconstruction was performed using vertebrate DNases and representative invertebrate DNases. In the maximum likelihood (ML) tree all the invertebrate sequences clustered together and were used as an outgroup to root the tree (Fig. 2a; Supplementary Fig. S1). Although basal chordates (e.g. ascidians and lancelets) can have more than one *DNase1* gene, they all cluster together in the invertebrate clade. These alternative sets of DNases likely derive from duplication events that occurred after separation from the vertebrate ancestor. Vertebrate DNases form eight distinct orthogroups clustered in four main branches. Human DNases are found in the lower (1/1L2) and in the upper (1L1/3) branches, while 1L4 and 1L5 branches do not comprise human orthologs. Clustering of human DNases into two clades is also evident in a heatmap based on pairwise sequence identity including only human DNases and their orthologs in chicken and zebrafish (Fig. 2b). Notably, DNase1/1L2 generally share higher amino acid sequence identity, suggesting a more recent separation of these genes.

Within the 1/1L2 branch, the DNase1 group appears to be paraphyletic with monophyletic DNase1L2 nested within it (Fig. 2a). The phylogenetic position of DNase1L2, together with its absence in fishes and amphibians, suggests that *DNase1L2* originated in amniotes. Within the DNase1 cluster, DNase1L2 has a sister group relationship with sauropsids DNase1, consistent with the observation that *Gg*DNase1 shares higher sequence identity with *Gg*DNase1L2 (57%) than with human DNase1 (*Hs*DNase1; 56%) (Fig. 2b).

Within the 1L1/3 branch, DNase1L3 and DNase1L1 show a low supported sister group relationship (Fig. 2a). Each of these groups has a sequence of lamprey in a basal branch, suggesting a separation before the agnathan-gnathostome split. The DNase1L3 cluster defines a single orthology group, at variance with the DNase1L1 cluster that contains two paralogous groups (DNase1L1 and DNase1L1L). The basal position of DNase1L1L and the distribution of the two orthogroups in vertebrates (see Fig. 1b) suggest duplication after the separation between cartilaginous and bony fishes, and subsequent loss of *DNase1L1L* in birds and mammals.

Basal to the 1L1/3 branch, DNase1L4 is a paraphyletic group with monophyletic DNase1L4-2 nested within it (Fig. 2a). The presence of *DNase1L4* in cartilaginous fishes suggests an early origin in vertebrate evolution, followed by gene loss in amniotes. The strongly supported phylogenetic position of DNase1L4-2 and its restricted gene distribution pattern (see Fig. 1b), places the origin of *DNase1L4-2* within the Neopterygii lineage.

The newly identified DNase1L5 orthogroup branches basal to DNase1L1/3/4 clusters, albeit with low bootstrap value (Fig. 2a). The well supported monophyly of the DNase1L5 group and the distribution of the *DNase1L5* gene in vertebrates (see Fig. 1b) suggest an early origin and loss in neopterygian fishes and amniotes.

### Sequence diversification of vertebrate DNase1 proteins

The members of the DNase1 family share high sequence similarity. Alignment of DNase1 family sequences show remarkable conservation of the catalytic residues involved in phosphodiester bond hydrolysis and magnesium binding (Fig. 2c), in accordance with homology models based on human DNase1 structure (Supplementary Fig. S2a). Conversely, no conservation of the actin binding residues was observed among these proteins, suggesting that actin inhibition is a recently acquired trait of placental mammals DNase1 (Fig. 2c; Supplementary Fig. S2b,c), even though it is not always conserved within the group^34^. All DNase1 family members, with the notable exception of DNase1L4/-2, contain a predicted N-terminal signal peptide and at least one pair of conserved cysteines potentially involved in disulfide bond formation (Fig. 2c; Supplementary Fig. S2d). Experimental evidence of disulfide bond formation comes from DNase1 crystallographic structures^35,36^ and from a very recent crystal structure of DNase1L3^37^. Of the three recognizable cysteine pairs, C4-C32 is found exclusively in DNase1L1, DNase1L1L, and DNase1L3 (Supplementary Fig. S2d), in agreement with the inferred phylogenetic relationship among these three groups; C101-C104 is present only in DNase1 and DNase1L5, although with few exceptions in DNase1 sequences (Supplementary Fig. S2c,d); C173-C209 is common to all DNase1 family members (Supplementary Fig. S2d). Interestingly, C173-C209 pair is present also in other chordates (e.g. lancelets) and in many invertebrate DNase1 sequences, whereas C101-C104 pair is not, indicating that the latter is a vertebrate-specific feature of DNase1/1L5 proteins that appeared after the divergence from chordates (Supplementary Fig. S2c). Given the inaccurate annotation of DNases in the protein sequence databases, the identification of a particular cysteine pair within a DNase1 sequence can aid the classification of the DNase1 family groups.

Proteins of the DNase1L5 group show the highest sequence similarity with DNase1 sequences, but with distinctive features (Supplementary Fig. S3a). In particular, the C-terminal regions differ for the presence of a cysteine-rich stretch in DNase1L5 sequences identified as a somatomedin B (SMB) domain (Supplementary Fig. S3b). In our ML tree, DNase1L5 sequences form a highly supported monophyletic group (see Fig. 2a) that branches near the invertebrate cluster and is almost equidistant from the DNase1/1L2 and DNase1L1/3/4 clusters (Supplementary Fig. S3c), in accordance with an early origin in the vertebrate lineage. The position in the phylogeny, together with the presence of *DNase1L5* in cartilaginous fishes, coelacanth (e.g. *L. chalumnae*; Sarcopterygii), reedfish (e.g. *E. calabaricus*; Actinopterygii) and amphibians, suggest independent losses in Neopterygii and amniotes (see Fig. 2b; Supplementary Fig. S4).

### Origin of *DNase1L2* by tandem duplication in terrestrial vertebrates

**Figure 3:**
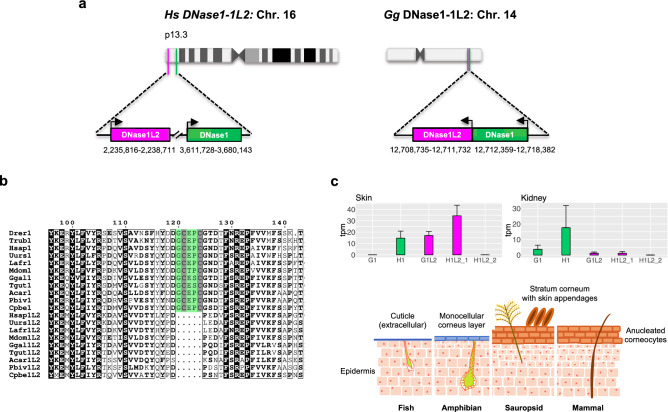
Origin of DNase1L2 by tandem duplication in terrestrial vertebrates. (**a**) Organization of *DNase1* and *DNase1L2* genes in chromosomes 16 and 14 of *Homo Sapiens* and *Gallus gallus*, respectively. (**b**) Portion of a multiple alignment of vertebrate DNase1 and DNase1L2 sequences showing the absence in DNase1L2 of a segment (green highlight) harboring a conserved cysteine pair in DNase1. (**c**) Upper panel: Gene expression levels of *DNase1* (1) and *DNase1L2* (1L2) in the skin and kidney of *H. sapiens* (H) and *G. gallus* (G) as derived from RNA-seq data analysis. The *G. gallus* skin samples were from the regenerating middle growth wing flight feather epithelium of the adult chicken^67^. H1L2_1: human transcripts corresponding to the DNase1L2-L isoform; H1L2_2: human transcripts corresponding to the DNase1L2-S isoform. Lower panel: schematic illustration showing the epidermis and skin appendages of vertebrates. The scheme is based on data from^41^.

DNase1L2 sequences form a well-separated monophyletic group within the DNase1 cluster (Fig. 2a; Supplementary Fig. S1). However, *Gg*DNase1L2 and *Gg*DNase1 share higher sequence identity (57%) compared to *Hs*DNase1L2 versus *Hs*DNase1 (48%; Fig. 2b), and *Gg*DNase1 has *Hs*DNase1L2, and not *Hs*DNase1, as the best hit. To understand the origin of DNase1L2, we first inspected the genomic loci in amniotes. In the chicken chromosome 14, *GgDNase1L2* and *GgDNase1* are located in tandem and separated by only ~ 630 bp , while in the human chromosome 16, *HsDNase1L2* and *HsDNase1* are separated by ~ 1370 kb and ~ 50 genes, albeit retaining the same orientation (Fig. 3a). Then, we investigated the multiple alignment of DNase1 and DNase1L2 sequences. Both mammal and sauropsid DNase1L2 include a fifteen-codon deletion corresponding to a peptide with a conserved cysteine pair (C101-C104) within a DNase1 loop (Fig. 3b; Supplementary Fig. S2d). This suggests that DNase1L2 originated by tandem duplication with deletion of the DNase1-specific loop before the divergence of the two amniote lineages (~ 330 Myr ago).

DNase1L2 was shown to degrade nuclear DNA derived from differentiating keratinocytes of human epidermis^38^ and murine skin appendages^6^. Therefore, we analyzed RNA sequencing (RNA-seq) data to confirm the expression of *DNase1L2* in human skin, finding high transcript levels (Fig. 3c, upper panel). We also observed expression of *DNase1* in human skin, even though to a lesser extent. In other tissues, including the kidney, where *DNase1* is normally expressed, *DNase1L2* displays a minimal expression (Fig. 3c, upper panel). Interestingly, we found that *DNase1L2* is also expressed in the feather epithelium of chicken (Fig. 3c, upper panel). Amniotes possess an outermost epithelial multilayer composed of anucleated corneocytes– terminally differentiated keratinocytes –the stratum corneum (Fig. 3c, lower panel)^39^, and different corneous appendages, such as hair and nails in mammals, and feathers and scales in birds and reptiles^40^. Conversely, the skin of fishes and amphibians is covered by a mucous cuticle and devoid of a stratified corneous layer^41^. Taken together, phylogenetic reconstruction and RNA-seq data suggest that soon after its origin, DNase1L2 acquired a specific function in the skin of terrestrial vertebrates.

### Origin of the DNase1L1/3 branch at the root of the vertebrates

The well-supported DNase1L1/3/4 cluster in the ML tree (Fig. 2a; Supplementary Fig. S1) suggests a basal position for the DNase1L4 group and a sister relationship between the DNase1L3 and DNase1L1 groups. It can be noticed that these DNase sequences harbor an additional C-terminal peptide that may have contributed to their functional diversification.

**Figure 4:**
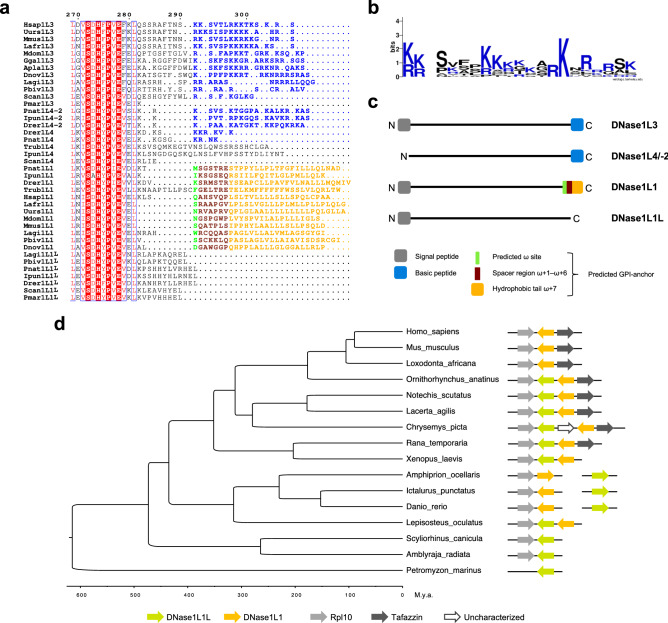
Origin of the DNase1L1/3 branch in the jawless vertebrate ancestor. (**a**) Portion of a multiple alignment of vertebrate DNase1L1, DNase1L1L, DNase1L3, DNase1L4 and DNase1L4-2 sequences featuring a different C terminus for each group. The alignment was manually edited to reflect the ω site prediction. The amino acid color code is as follows: blue for basic residues, green for the predicted ω site, brown for the spacer region (from ω + 1 to ω + 6), orange for the hydrophobic tail (to ω + 7). (**b**) Frequency plot of the aligned C-terminal basic peptides from 17 sequences of vertebrate DNase1L3, DNase1L4 and DNase1L4-2. (**c**) Scheme of vertebrate DNase1L1, DNase1L1L, DNase1L3, and DNase1L4/-2 sequences, showing the diversification of N- and C-terminal regions. (**d**) Chronogram of vertebrate phylogeny derived from TimeTree. Genomic organization of the *DNase1L1* and *DNase1L1L* loci in the different species is displayed at the terminal nodes, showing synteny with *Rpl10* in gnathostomes and with *Tafazzin* in tetrapods.

DNase1L3 proteins contain a positively charged C-terminal peptide (Fig. 4a,b), which has been shown to be essential for the digestion of nucleosomal DNA of circulating microparticles released from apoptotic cells^42^. The lack of this peptide in DNase1L3 of lamprey suggests that this trait emerged in gnathostomes (Fig. 4a). The same C-terminal peptide is present in DNase1L4 sequences, with some exceptions (Fig. 4a,b). The DNase1L4 proteins, which are absent in humans, predominate in Neopterygii; conversely, DNase1L3 was lost by the main bony fish clade (Euteleosteomorpha), while it is maintained in Otomorpha with the notable exception of *Danio rerio* (Supplementary Fig. S4). *DNase1L4-2* likely originated by an early tandem duplication of *DNase1L4* in bony fishes after the separation from Sarcopterygii, and was independently lost in several lineages (Supplementary Fig. S4).

DNase1L1 sequences contain a hydrophobic C-terminal peptide for attachment to the plasma membrane via a GPI anchor (Fig. 4a), which has been shown to be conserved in all mammalian DNase1L1 proteins^43,44^. Prediction of GPI-anchor targeting signals applied to the full set of DNase sequences, confirmed the C-terminal signal in mammalian DNase1L1, and further revealed the conservation of the GPI-anchor signal and ω site in vertebrate DNase1L1 (Fig. 4a,c). Conversely, DNase1L1L sequences do not contain a GPI-anchor signal and yet show conservation of the C-terminal residues “EL” (Fig. 4a). These distinct C-terminal ends can be used to discriminate between DNase1L1 and DNase1L1L proteins (Fig. 4c).

In lamprey and cartilaginous fish, the presence of DNase1L1L, but not DNase1L1, suggests that *DNase1L1* originated from *DNase1L1L* by tandem duplication in a bony fish ancestor of Actinopterygii and Sarcopterygii (~ 450 Myr ago; Supplementary Fig. S4). *DNase1L1L* and *DNase1L1* are still found in tandem in some vertebrates (Fig. 4d). However, the retention of both paralogs was observed only in fishes, amphibians, reptiles, and non-placental mammals, while in birds and placental mammals *DNase1L1L* was lost (see Fig. 1b). In tetrapods, *DNase1L1* lies head-to-head with *Tafazzin*, whose product is a transacylase acyltransferase involved in membrane lipid remodeling.

### *DNase2b* is the ancestor of the DNase2 family in vertebrates

**Figure 5:**
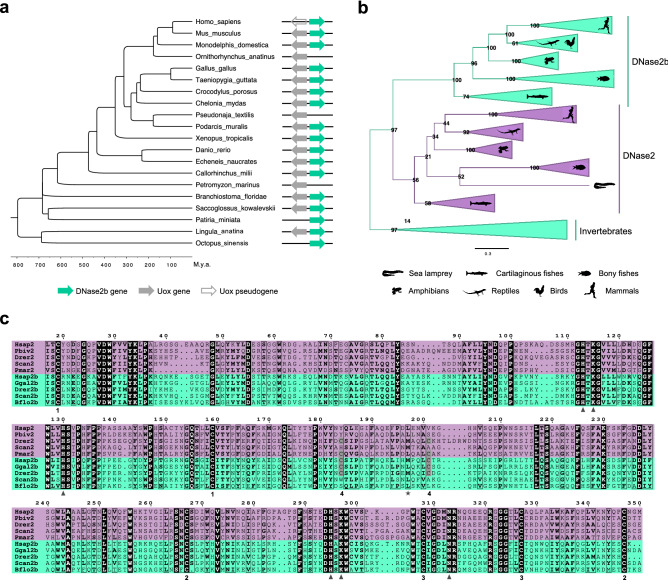
Origin and conservation of DNase2 family in vertebrates. (**a**) Chronogram of Metazoa phylogeny derived from TimeTree (for species dating, *Lingula adamsi* and *Octopus vulgaris* were used instead of *Lingula anatina* and *Octopus sinensis*, respectively). Genomic organization of the *DNase2b* locus in the different species is displayed at the terminal nodes, showing extensive conservation of *DNase2b-Uox* synteny. Exceptions are represented by species in which one of the two genes is missing. (**b**) Maximum-likelihood phylogeny of 86 DNase2 family proteins inferred with the LG substitution model. Tree branches were collapsed into subgroups representing DNase2 and DNase2b in different groups of organisms. Invertebrate sequences were used to root the tree. Bootstrap values are shown at the internal nodes. Scale bar, substitution/site. The full tree is shown in Supplementary Fig. S6. (**c**) Portion of a multiple alignment of DNase2 family sequences from selected species. The catalytic residues of the PLD domains are indicated with triangles; the conserved cysteines pairs forming disulfide bonds are indicated with the same number, and the cysteines pair conserved only in DNase2b is also highlighted in dark gray; the first methionine of DNase2b-S is indicated with a star (see Fig. 6). The full alignment is shown in Supplementary Fig. S7.

The origin of the DNase2 family was first addressed with synteny analysis. *DNase2b* lies head-to-head and shares a bidirectional promoter with the *Uox* gene. This organization is observed in nearly all chordates and even in Hemichordata (i.e. *Saccoglossus kowalevskii*) and Brachiopoda (i.e. *Lingula anatina*), suggesting that it existed at the protostome-deuterostome divide (Fig. 5a). Indeed, *DNase2b* was already present in basal Eumetazoa, in accordance with our HMM-based search in complete genomes (see Fig. 1b). Synteny conservation of *DNase2b* and the emergence of two separated *DNase2* genes in vertebrates only, provide clear evidence that *DNase2b* is the ancestor of the DNase2 family.

Phylogenetic reconstruction of the DNase2 family was performed using DNase2b and DNase2 of vertebrate species, and DNase2b of invertebrate species. Similarly to the DNase1 tree, the DNase2 ML tree showed a highly supported separation between vertebrate and invertebrate sequences; therefore the invertebrate DNase2b sequences were used as an outgroup to root the tree (Fig. 5b; Supplementary Fig. S5). Within the vertebrate clade, DNase2 and DNase2b are monophyletic sister groups; the only lamprey DNase2 sequence clusters within the DNase2 group, basal to the bony fish clade. At variance with the DNase2 group, the DNase2b cluster shows a strongly supported branching of the different vertebrate groups. Notably, a longer branch leads to vertebrate DNAse2b likely reflecting an accelerated evolutionary rate. The presence of *DNase2* in lamprey (see Fig. 1b) suggests that *DNase2* originated before the agnathan-gnathostome separation; *DNase2b* was likely lost in agnathans after their divergence from jawed vertebrates.

DNase2 and DNase2b show considerable identity in their amino acid sequence (~ 40%), suggesting that they share a common structure and a common catalytic strategy as evidenced by the two conserved Phospholipase D (PLD) domains, each harboring a HxK motif, and by the three pairs of conserved cysteines (Fig. 5c; Supplementary Fig. S6). DNase2b has an additional pair of conserved cysteines that is also found in DNase2 of bony fishes and lamprey. The absence of this trait in the sequences of amphioxus and other invertebrates, indicates that it is a vertebrate-specific acquisition. Furthermore, a N-terminal signal peptide was predicted in all DNase2 and DNase2b sequences (Supplementary Fig. S6), suggesting that the lysosomal localization of DNase2 and DNase2b proteins^45,46^ could have been maintained (possibly with the exception of the DNase2b short isoform; see below) since their origin.

### Structural modification of DNase2b in placental mammals

**Figure 6:**
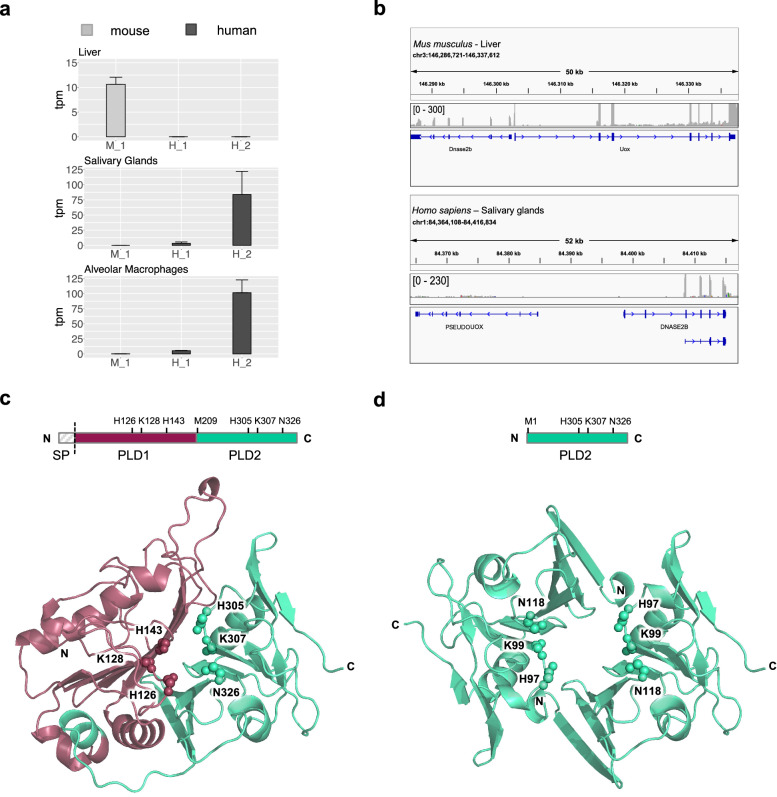
Expression and structural organization of the two DNase2b isoforms. (**a**) Gene expression levels of *DNase2b* in the liver, salivary glands, and alveolar macrophages of *M. musculus* (M) and *H. sapiens* (H) derived from RNA-seq data analysis. H_1: *H. sapiens* transcripts corresponding to the long isoform; H_2: *Homo sapiens* transcripts corresponding to the short isoform. (**b**) Reads mapping to *DNase2b* showing expression of the long isoform in mouse liver and expression of the short isoform in human salivary glands. (**c**) Upper panel: DNase2b domain composition; the dashed line indicates the signal peptide (SP) cleavage; lower panel: 3D model of DNase2b pseudodimeric structure with the catalytic residues shown in spheres and labeled. The model was downloaded from the AlphaFold database (AF-Q8WZ79-F1) and manually edited to remove the signal peptide. (**d**) Upper panel: DNase2b-S domain composition; lower panel: 3D model of DNase2b-S homodimeric structure with the catalytic residues shown in spheres and labeled. The model was obtained by de novo AlphaFold prediction assuming a homodimeric assembly.

The conservation of a bidirectional promoter between the divergently transcribed *DNase2b* and *Uox* genes, results in their coordinated expression in the liver of mammals. In humans, loss of *DNase2b*-*Uox* association following *Uox* pseudogenization has been associated with the shift of *DNase2b* expression from liver to salivary glands^22^. A short isoform is produced through an alternative transcription start site (TSS) located within the second intron of human *DNase2b*^14^. Our analysis of RNA-seq data confirmed that *DNase2b* is expressed in mouse but not in human liver, and revealed that the short isoform of *DNase2b* is highly expressed in human salivary glands and alveolar macrophages as the main isoform (Fig. 6a). Mapping of RNA-seq reads confirmed the presence of a TSS in the second intron of human *DNase2b*, with respect to the upstream TSS observed in the mouse sequence (Fig. 6b). *DNase2b* was shown to be responsible for the degradation of nuclear DNA in mouse lens fibers^7^. RNA-seq data confirmed the presence of abundant *DNase2b* transcripts (corresponding to the long isoform) in mouse lens, but not in human lens (Supplementary Fig. S7a). Although we did not find transcripts corresponding to the short DNase2b isoform in the analyzed mouse tissues, we found them in non-primate mammals (Supplementary Fig. S7b,c), indicating that this isoform predates *Uox* pseudogenization. A multiple sequence alignment of vertebrate DNase2b sequences showed that the first methionine (M209) of *Hs*DNase2b short isoform, is highly conserved in mammals with the exception of marsupials and some rodents (Supplementary Fig. S7d). Sequences of rodents, particularly *Mus musculus* and *Rattus norvegicus*, diverge from the sequences of placental mammals by several point mutations at conserved positions. This is reflected in the odd position and long branches of rodent DNase2b proteins in the phylogeny (see Fig. 5b and Supplementary Fig. S5).

To get insights into the functional significance of the two different *DNase2b* transcripts in mammals, we analyzed the sequence and domain organization of the long and short protein isoforms (Fig. 6c,d). The polypeptide chain corresponding to the long DNase2b isoform (335 aa) is organized into two PLD domains (PLD1 and PLD2). Each domain contains a catalytic triad (H, K, H/N) involved in the phosphodiester bond hydrolysis. The polypeptide chain corresponding to the short N-truncated isoform (DNase2b-S; 153 aa), coincides with the PLD2 domain of the long isoform. The AlphaFold^47,48^ model of DNase2b (there is no experimentally determined structure for any of the eukaryotic DNase2) showed that the bidomain protein folds into a pseudodimer with the active site located at the PLD1-PLD2 interface (Fig. 6c). Accordingly, we modeled de novo the structure of DNase2b-S assuming a homodimeric quaternary structure (Fig. 6d). The predicted AlphaFold model shows an overall similar structure to that of DNase2b with the active site located at the PLD2 dimer interface, suggesting that DNase2b-S can be catalytically active despite the absence of one catalytic domain (PLD1). The large number of mutations observed in *M. musculus* DNase2b (Supplementary Fig. S7e) may indicate a relaxed selective pressure due to the loss of the short isoform as suggested by lack of conservation of the starting methionine and absence of the corresponding transcripts in RNA-seq data. Our results suggest that DNase2b is conserved among vertebrates, while DNase2b-S is specific to placental mammals, although it is probably absent in rodents.

## Discussion

**Figure 7:**
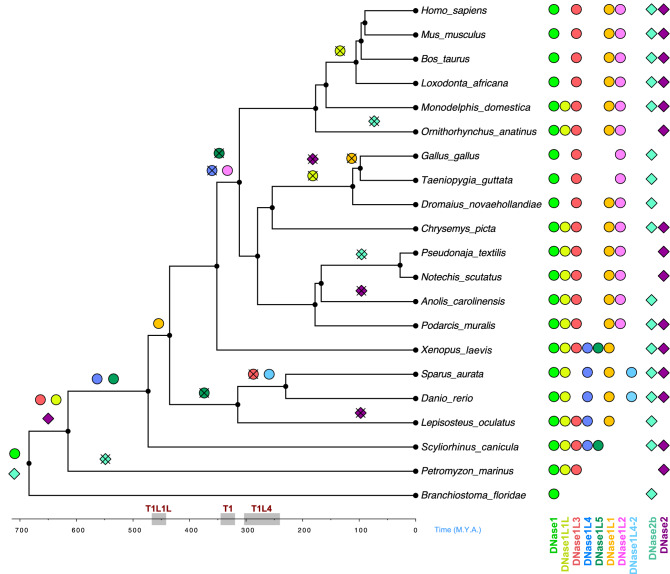
Evolutionary history of DNase1 and DNase2 families. Gene gains: *DNase1* (circles); *DNase2* (diamonds). Gene losses: *DNase1* (crossed-out circles); *DNase2* (crossed-out diamonds). Data inferred from protein phylogeny and gene distribution are mapped onto a chordate chronogram. Species relationships and divergence times are from TimeTree (for species dating, *Callorhinchus milii* and *Xenopus tropicalis* were used instead of *Scyliorhinus canicula* and *Xenopus laevis*, respectively). The DNase gene repertoire of the 21 chordate species is displayed at the terminal nodes. Time intervals of tandem duplications involving *DNase1L1L* (T1L1L), *DNase1* (T1), and *DNase1L4* (T1L4) are indicated with gray boxes on the time scale.

With the aim to understand the significance of the human DNase repertoire, we investigated the origin and diversification of DNase1 and DNase2 families in vertebrate evolution. DNase genes outside Metazoa are restricted to a few clades of unicellular eukaryotes, and to the most basal clades of fungi and plants (see Fig. 1b). The basal position of these sequences in the phylogeny (Supplementary Fig. S8a,b) is in keeping with a scenario of vertical transmission and gene loss in different lineages. Within metazoans, invertebrates have a minimal DNase repertoire, including one *DNase1* and one *DNase2* gene, whereas vertebrates possess an expanded repertoire including eight *DNase1* and two *DNase2* genes. Our phylogenetic reconstructions of the DNase1 and DNase2 families, in conjunction with the examination of multiple sequence alignments and synteny analysis, shed light on the origin and relatedness of DNase1 and DNase2 orthogroups. We note that although the bootstrap values for the monophyly of individual families (with the exception of DNase1L1) are high, the relationships among the different families are often less supported by bootstrap. This could be explained by a diversification of DNase families over relatively short timescales (evolutionary radiation). A further reason may be related to the low sequence diversification of the different families, which maintain a similar catalytic activity notwithstanding the functional specialization. Either way, the low support received from some relationships between DNase families introduces a note of caution in the interpretation of the inferred phylogeny. The proposed evolutionary history of the two protein families is schematized in Fig. 7:

i. Within the DNase2 family, DNase2b was already present in invertebrates as evidenced by the genomic association between *DNase2b* and *Uox* that predates the origin of two separated *DNase2* (*DNase2b* and *DNase2*) genes. Within the DNase1 family, a DNase1 predating the split among the most basal 1/1L1/1L3 groups was present in invertebrates.
ii. A first expansion of the DNase repertoire in vertebrates involved *DNase2, DNase1L3*, and *DNase1L1L* which originated before the divergence of the jawless and jawed vertebrate lineages. A second expansion involved *DNase1L4* and *DNase1L5*, which could have originated in jawed vertebrates, even though an earlier origin and subsequent loss in jawless vertebrates cannot be ruled out. It can be hypothesized that this expanded set of genes arose from the two rounds of genome duplications at the root of the vertebrates (Simakov et al. 2020).
iii. The remaining *DNase1* genes originated by tandem duplication events. *DNase1L1* originated from *DNase1L1L* in bony vertebrates (T1L1L); *DNase1L2* originated from *DNase1* in amniotes (T1); *DNase1L4-2* originated from *DNase1L4* in bony fishes (T1L4).
iv. Several gene losses occurred in both families: *DNase1L3* and *DNase1L4* were lost in bony fishes and amniotes respectively; *DNase1L1L* was lost independently in birds and placentals; *DNase1L1* was lost in Neognathae birds; *DNase1L5* was lost independently in bony fishes and amniotes; *DNase2b* was lost in agnathans, snakes and monotremes; *DNase2* was lost in birds.

Within the DNase1 family, the 1/1L2 branch DNases have been maintained in all vertebrate organisms since their origin. *DNase1* is expressed in the human digestive system, but also in many other tissues (Supplementary Table S1). It was shown to be broadly expressed in mammals, particularly in many organs of the gastrointestinal and urogenital tract^50,51^. Because of this tissue distribution, DNase1 is classically regarded as a digestive enzyme, although its involvement in the degradation of intravascular NETs released from neutrophils during inflammation has been established^18^. *DNase1L2* is the most recent member of the family as it originated in amniotes, and seems to be a skin-specific gene both in humans and birds (see Fig. 3c, upper panel; Supplementary Table S1). Notably, DNase1L2 is the only DNase1 family enzyme with acidic pH optimum (5.5–6)^14^, a feature that can be an adaptation to the acidic pH of the epidermis. In vivo studies demonstrated that DNase1L2 is required for nuclear DNA degradation during the formation of the stratum corneum and skin appendages in mice^6,52^, a process known as cornification^41^. The development of the stratum corneum and skin appendages serving as a protective skin barrier has been fundamental for vertebrate terrestrialization^53^. Arguably, skin cornification in amniotes determined the need to clear abundant extracellular DNA released by differentiating keratinocytes. Our phylogenetic and RNA-seq data analyses suggest that the acquisition of a skin-specific DNase constitutes an innovation of terrestrial vertebrates related to the formation of the stratum corneum and skin appendages. As such, *DNase1L2* can be thought as a gene functional to the adaptation of vertebrate life on land.

The evolution of DNases was accompanied by architectural diversification of the N-terminal and C-terminal protein regions that are conserved in all vertebrate sequences and entailed functional specialization (see Fig. 4). A C-terminal basic peptide is a conserved feature in DNase1L3 proteins (see Fig. 4a), providing the capability to digest microparticles-associated DNA that is abundantly produced by the turnover of myeloid cells^42^. Accordingly, DNase1L3 is synthesized by myeloid cells and contributes to the homeostasis of the hematopoietic system and immune function^54^. Notably, RNA-seq data show very high *DNase1L3* expression levels in spleen and liver (Supplementary Table S1). Sequence comparison suggests that DNase1L3 acquired the capability to degrade membrane-encapsulated DNA in jawed vertebrates. This feature may be shared by DNase1L4 and DNase1L4-2, which also harbor a C-terminal basic region. This hypothesis is further supported by the different distribution of the three endonucleases in vertebrates (see Fig. 1b). However, DNase1L4/-2 may act inside the cell because it lacks the N-terminal signal peptide (see Fig. 2c).

In DNase1L1, the C-terminal GPI-anchor signal allows anchoring of the protein to the outer layer of the cell membrane as an ectoenzyme. This unique property was gained by DNase1L1 soon after its origin because the GPI-anchor signal is found in DNase1L1 of bony vertebrates, but not in DNase1L1L proteins (see Fig. 4a). Membrane-anchoring of DNase1L1 is crucial for preventing endocytosis-mediated transfer of foreign DNA^44^ and permits the localization of the endonuclease within podosomes, protrusive membrane structures with adhesive and degradative functions formed by migrating cells, such as immune cells, macrophages and dendritic cells^19^. According to RNA-seq data, *DNase1L1* is expressed at high levels in skeletal and cardiac muscle cells, and at basal levels in several other tissues, consistent with its activity in different types of cells (Supplementary Table S1). The maintenance of the GPI-anchor signal in bony vertebrates suggests that DNase1L1 has a conserved role in the muscular tissue and immune system. However, the loss of the gene in birds remains unexplained.

Very little is known about the role of DNase1L1L which does not show any known functional motif at the C-terminal end. *DNase1L1L* was reported to be uniquely expressed in zebrafish lens and responsible for nuclear DNA degradation during lens development^55^.

A C-terminal SMB domain is a conserved feature of DNase1L5 sequences (see Supplementary Fig. S3a,b). This cysteine-rich domain of uncertain function (pfam id: PF01033) is found in different proteins, usually in association with phosphodiesterase and endoribonuclease domains. Members of the DNase1L5 group have been previously characterized in amphibians, but have been referred to as DNase1, in spite of the very distant position from DNase1 proteins of other vertebrates^32,56,57^. The SMB domain was shown to be required for the recombinant production of an active DNase^56^.

The two DNase2 family members do not exhibit remarkable differences in their protein sequences (see Fig. 5c). On the contrary, gene expression of *DNase2* and *DNase2b* differs markedly. *DNase2* is abundantly expressed in the majority of human tissues (Supplementary Table S1). The same ubiquitous expression was observed in mammals^22,58^, and accordingly, DNase2 has been indicated as the primary enzyme responsible for the phagocyte-mediated DNA degradation in lysosomes^59^. Furthermore, DNase2 present in macrophages is required for efficient erythropoiesis in fetal liver, specifically for the enucleation of erythrocytes precursors^5^. DNase2 acquired this particular role in mammals because in non-mammalian vertebrates mature erythrocytes are nucleated.

*DNase2b* is specifically expressed in the liver of mammals and associated with *Uox* expression (see Fig. 6a,b). According to RNA-seq data analysis, we also found *DNase2b* expression independent from *Uox* expression in other mammalian tissues, such as lens and salivary glands (see Supplementary Fig. S8a,c). Humans and apes lost hepatic expression of *DNase2b* following *Uox* pseudogenization. We observed expression of *DNase2b* in human salivary glands and alveolar macrophages, but, in contrast with previous evidence^7^, we did not find expression in RNA-seq data from human lens. Importantly, at variance with the long isoform observed in mammalian liver and lens, a short N-truncated isoform is the predominant one in the analyzed human tissues (see Fig. 6a,b). By using de novo prediction of 3D structure we show that the product of the short isoform, DNase2b-S, can fold into a homodimeric assembly that bears a close similarity to the DNase2b pseudodimeric structure, despite the lack of one PLD domain (see Fig. 6c,d). Because we found transcripts corresponding to the short isoform in non-primate mammals, but not in mice, and we observed that the first methionine of DNase2b-S is conserved in placental mammals, but not in rodents, we propose that the short isoform of *DNase2b* originated in placental mammals and was lost in rodents.

In conclusion, our results reveal an expansion of the *DNase* gene families during the evolution of vertebrate organisms. The appearance of novel cell types in the hematopoietic system and novel tissue structures such as the stratum corneum was accompanied by the functional differentiation of DNase activities to meet the requirement for the clearance of DNA debris in complex multicellular organisms.

## Methods

### Classification of DNase1 and DNase2 family proteins

All human members of DNase1 and DNase2 families and homologs annotated for other vertebrate species were downloaded from Ensembl (Release 104). In parallel BlastP searches were carried out to check the corresponding sequences in NCBI reference proteomes. To expand searches to taxa that did not produce significant hits in the BlastP searches and to avoid false negatives, additional tBLASTn searches against reference genomes were performed. Grouping into families was performed based on phylogenetic analysis, functional domain composition, and synteny analysis. For each family, all sequences were visually inspected to remove incomplete or ambiguous proteins. Because many sequences had incorrect names, we modified the header of the fasta file according to our classification.

### Phylogenetic analysis

The obtained members of DNase1 and DNase2 families were aligned using ClustalO with default settings at the EMBL-EBI webportal (https://www.ebi.ac.uk/Tools/msa/clustalo/)^60^. Maximum-Likelihood (ML) trees were constructed with PhyML v3.0 (http://www.atgc-montpellier.fr/phyml/)^61^ using the LG substitution model, or the WAG/JTT substitution model as set by the automated model selection procedure^62^. Nodal support was estimated by bootstrap analysis with 100 replicates. Figures were created with FigTree v1.4.3 (https://github.com/rambaut/figtree/).

### HMM-based search in complete genomes

Hidden Markov models (HMM)^63^ were exploited for the search of DNase protein sequences in complete genomes of vertebrate and invertebrate organisms. For each DNase1 and DNase2 family group, 10–20 sequences corresponding to bona fide orthologs were aligned with ClustalX at default parameters. From the multiple alignments 10 HMM profiles corresponding to the 8 DNase1 and 2 DNase2 orthogroups were built with HMMER. These HMM profiles were used to scan representative genomes of vertebrate and invertebrate species with an E-value threshold of 10^–20^. Individual proteins were assigned to DNase family groups according to their best match with the HMM profiles. The figure of gene distribution map across the eukaryote phylogeny was drawn using the R language and the taxizedb (https://docs.ropensci.org/taxizedb/) and ggtree^64^ libraries.

### 3D structure prediction

Homology modeling was performed using SWISS-MODEL (https://swissmodel.expasy.org)^65^. De novo prediction of 3D structure was carried out with AlphaFold (https://colab.research.google.com/github/sokrypton/ColabFold/blob/main/AlphaFold2.ipynb)^66^. Analysis of the protein structures and images of atomic models were performed with PyMOL (The PyMOL Molecular Graphics System, Version 1.3 Schrödinger, LLC.).

### RNA-seq data analysis

Publicly available RNA-seq data from selected tissues of *Homo sapiens*, *Mus musculus*, *Canis lupus familiaris*, and *Gallus gallus* (Supplementary Table S2) were downloaded from the NCBI Sequence Read Archive (SRA) database to quantify RNA abundance. Low-quality reads were removed using FASTP software v0.19.5 (https://github.com/OpenGene/fastp/) with default parameters. From cleaned FASTQ files, pseudoalignment and transcript quantification was obtained using the Kallisto software v0.46.2 (https://pachterlab.github.io/kallisto/). For the quantification of expression of the gene of interest, tpm values of transcripts encoding the same protein were summed. Bar plots were created using the ggplot2 R package. Three biological replicates were considered for each tissue type. To map reads into target genes, cleaned reads were aligned to the reference genome using HISAT2 software v2.2.1 (http://daehwankimlab.github.io/hisat2/). Coordinated-sorted bam files and bai files were used as input for the IGV (Integrative Genomic Viewer) software v2.11.0 (https://software.broadinstitute.org/software/igv/) to obtain transcript mapping images.

## Supplementary Information

Supplementary Information 1.

Supplementary Information 2.

Supplementary Information 3.

Supplementary Information 4.

Supplementary Information 5.

## Data Availability

Raw data generated in this study are deposited in the Harvard dataverse repository (https://doi.org/10.7910/DVN/HRJXNE).
