# Supplementary material for: Origin and significance of the human DNase repertoire
Source: Sci Rep. 2022 Jun 20;12:10364. doi: 10.1038/s41598-022-14133-w (PMC9208542; doi:10.1038/s41598-022-14133-w)
Supplement: Supplementary file 1 — Supplementary Information 1. [file 41598_2022_14133_MOESM1_ESM.pdf]

Supplementary Figure S1. Maximum-likelihood phylogeny of 237 DNase1 family proteins inferred with the LG substitution model.

Supplementary Figure S2. Structural features of DNase1 proteins.

Supplementary Figure S3. Identification of DNase1L5 proteins containing a C-terminal SMB domain.

Supplementary Figure S4. Chronogram of jawed fish phylogeny derived from TimeTree.

Supplementary Figure S5. Maximum-likelihood phylogeny of 86 DNase2 family proteins inferred with the LG substitution model.

Supplementary Figure S6. Multiple alignment of DNase2 family sequences from selected species.

Supplementary Figure S7. Expression and conservation of DNase2b isoforms.

Supplementary Figure S8. Phylogeny of DNases in eukaryotes.

Supplementary Table S1. Expression profile of DNase1 and DNase2 from two human RNA-seq datasets from NCBI.

Supplementary Table S2. Sequence read archive IDs of the RNA-seq data analyzed for DNase gene expression.

Supplementary Data 1. DNase1 and DNase2 sequence information in tabulated form.

Supplementary Data 2. DNase1 and DNase2 sequences in fasta format.

Supplementary Data 3. Multiple alignment in interleaved phylip format of DNase1 family proteins used for the phylogenetic reconstruction.

Supplementary Data 4. Multiple alignment in interleaved phylip format of DNase2 family proteins used for the phylogenetic reconstruction.

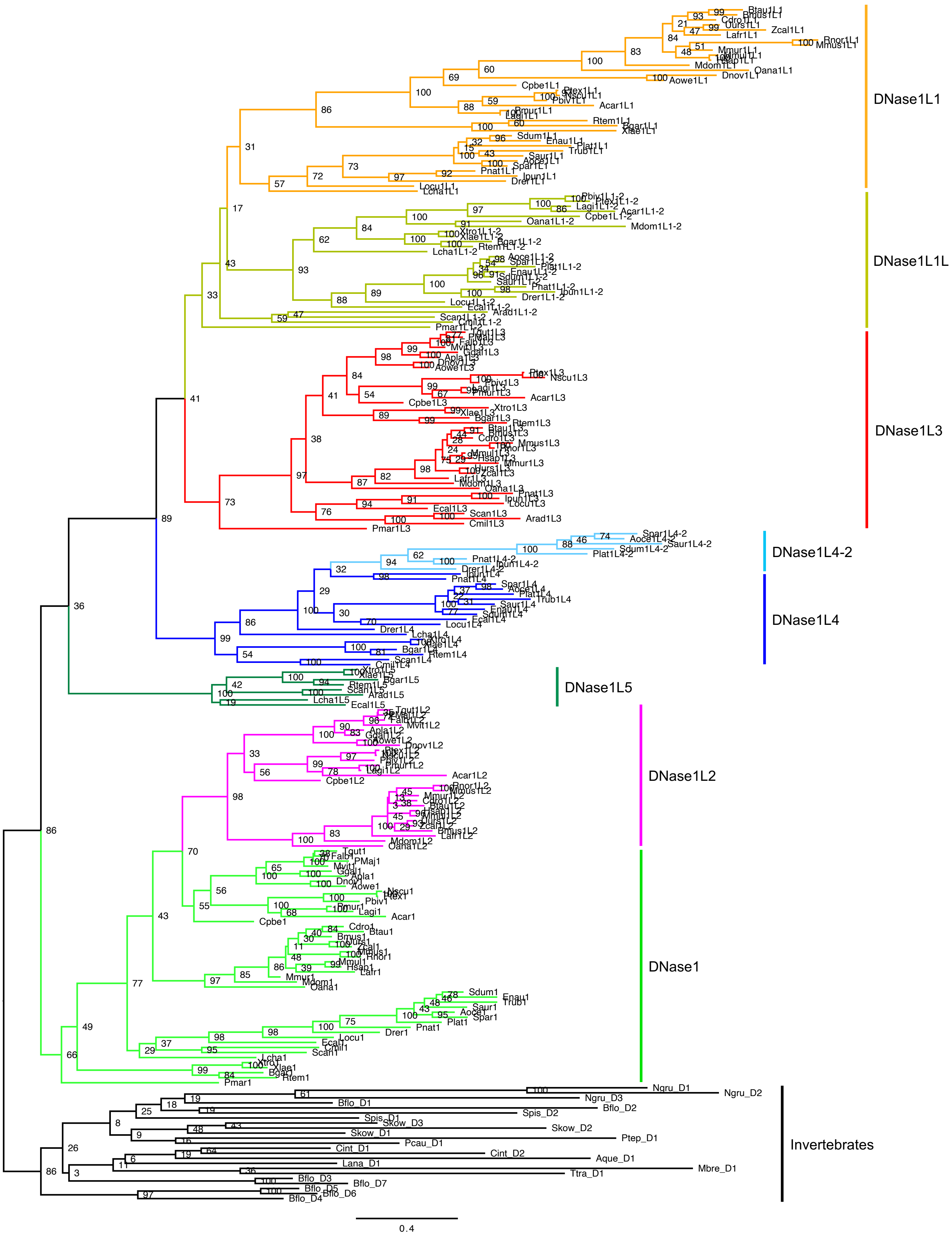

Figure S1. Maximum-likelihood phylogeny of 237 DNase1 family proteins inferred with the LG substitution model. Sequences are labeled with the abbreviated taxon name followed by the DNase1 family member identifier according to our classification. Invertebrate sequences were used to root the tree. Bootstrap values are shown at the internal nodes. Scale bar, substitution/site.

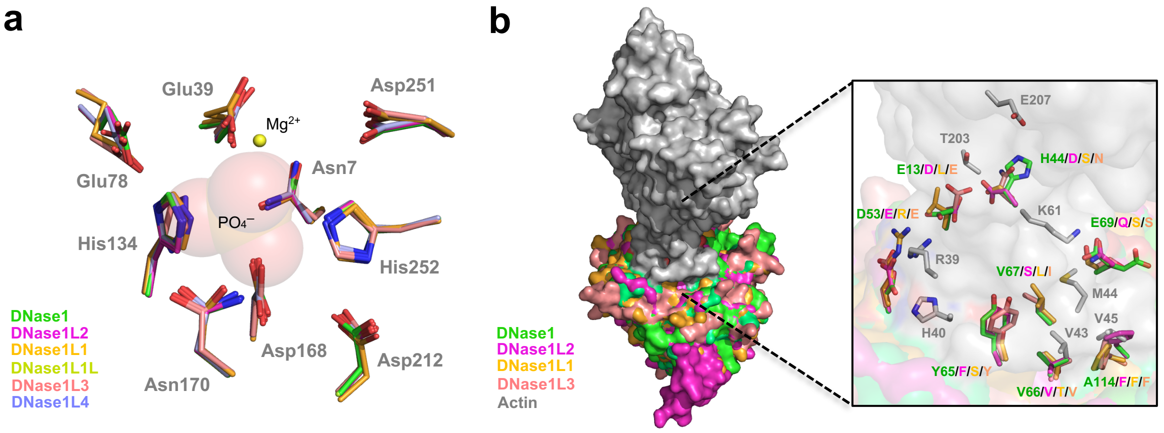

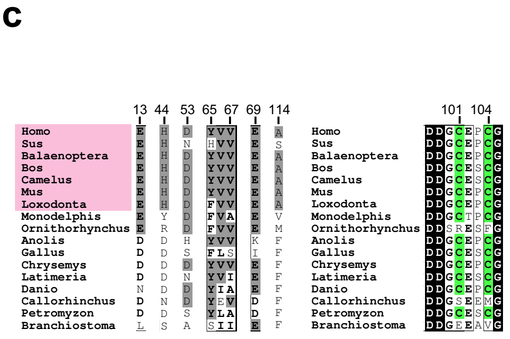

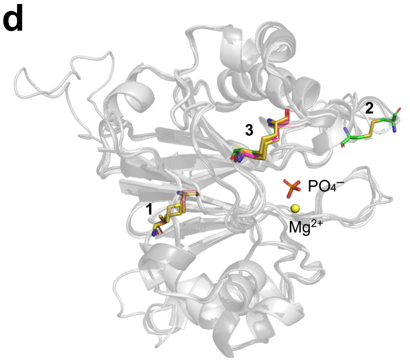

Protein	Cysteine pair		
	1	2	3
DNase1 (1ATN)	-	101-104	173-209
DNase1L2	-	-	173-209
DNase1L1	4-32	-	173-209
DNase1L1L	4-32	-	173-209
DNase1L3	4-32	-	173-209
DNase1L4	-	-	-
DNase1L4-2	-	-	-
DNase1L5	-	101-104	173-209

Figure S2. Structural features of DNase1 proteins. (**a**) Superposition of the active sites of human DNase1 structure (4AWN; green carbons) and the homology models of DNase1L1 (orange carbons), DNase1L1L (lime carbons), DNase1L2 (pink carbons), DNase1L3 (salmon carbons), and DNase1L4 (light blue carbons) obtained with 4AWN as template, showing strict conservation of active site residues. This conservation is also observed in groups not included in the structure superimposition (i.e. DNase1L4-2 and DNase1L5). Bound magnesium and phosphate groups are shown in yellow and orange, respectively. The residue numbering refers to 4AWN as shown in **Fig. 2c** and it is maintained in the following (**b**) and (**c**) panels. (**b**) Superimposition of the human DNase1 surface structure (4AWN; green) with the homology models of DNase1L1 (orange), DNase1L2 (pink), and DNase1L3 (salmon), and the actin-bovine DNase1 complex (1ATN; gray), showing the DNase-actin interaction. Key residues at the actin binding interface are shown in sticks with labels in the inset. (**c**) Aligned sequences of DNase1 from chordates showing conservation of actin binding residues in placental mammals (pink highlight; left panel), and conservation of a putative disulfide bond in vertebrates (green highlight; right panel). The residue numbering is as in (**b**) and (**d**) panels. (**d**) Left panel: superposition of the bovine DNase1 structure (4ATN; gray cartoon and green carbon sticks) and the same homology models shown in (**a**). Whole structures are shown in gray cartoon and conserved cysteine pairs likely involved in disulfide bonds are shown in sticks colored as in (**b**). Bound magnesium and phosphate groups are shown in yellow and orange, respectively. Right panel: conserved cysteine pairs in DNase1 family proteins. The residue numbering refers to 4AWN.

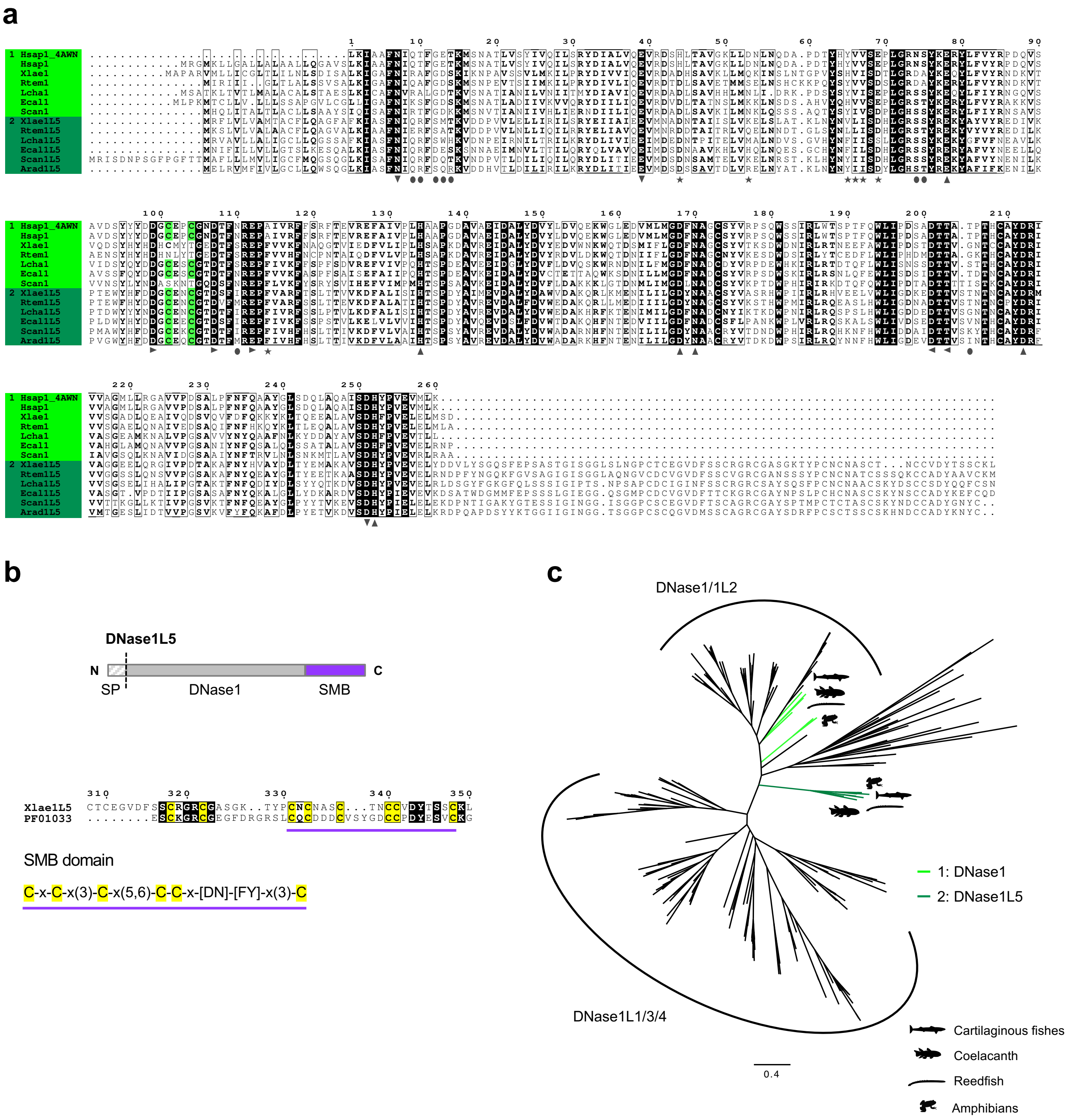

Figure S3. Identification of DNase1L5 proteins containing a C-terminal SMB domain. (**a**) Multiple alignment of DNase1 (group 1; green) and DNase1L5 (group 2; dark green) proteins from vertebrate species. Remarkable residues are indicated as follows: catalytic, up triangles; Mg^{2+} binding, down triangles; Ca^{2+} binding in site I, right triangles; Ca^{2+} binding in site II, left triangles, actin binding, stars; DNA binding, circles; pairs of conserved cysteines C101-C104 are highlighted in green. (**b**) Upper panel: DNase1L5 domain composition; the dashed line indicates signal peptide (SP) cleavage; lower panel: portion of a pairwise alignment between *X. laevis* DNase1L5 and the consensus sequence containing the somatomedin B domain (SMB; pfam id: PF01033) showing conservation of the eight cysteines (yellow highlight) possibly involved in disulfide bond formation; the Prosite pattern for the SMB domain is shown below the alignment. (**c**) Unrooted maximum-likelihood phylogeny of 237 DNase1 family proteins, showing the position of DNase1 (group 1; green) and DNase1L5 (group 2; dark green) sequences displayed in the alignment in (**a**). The phylogeny is the same shown in Fig. 2a and in Supplementary Fig. S1.

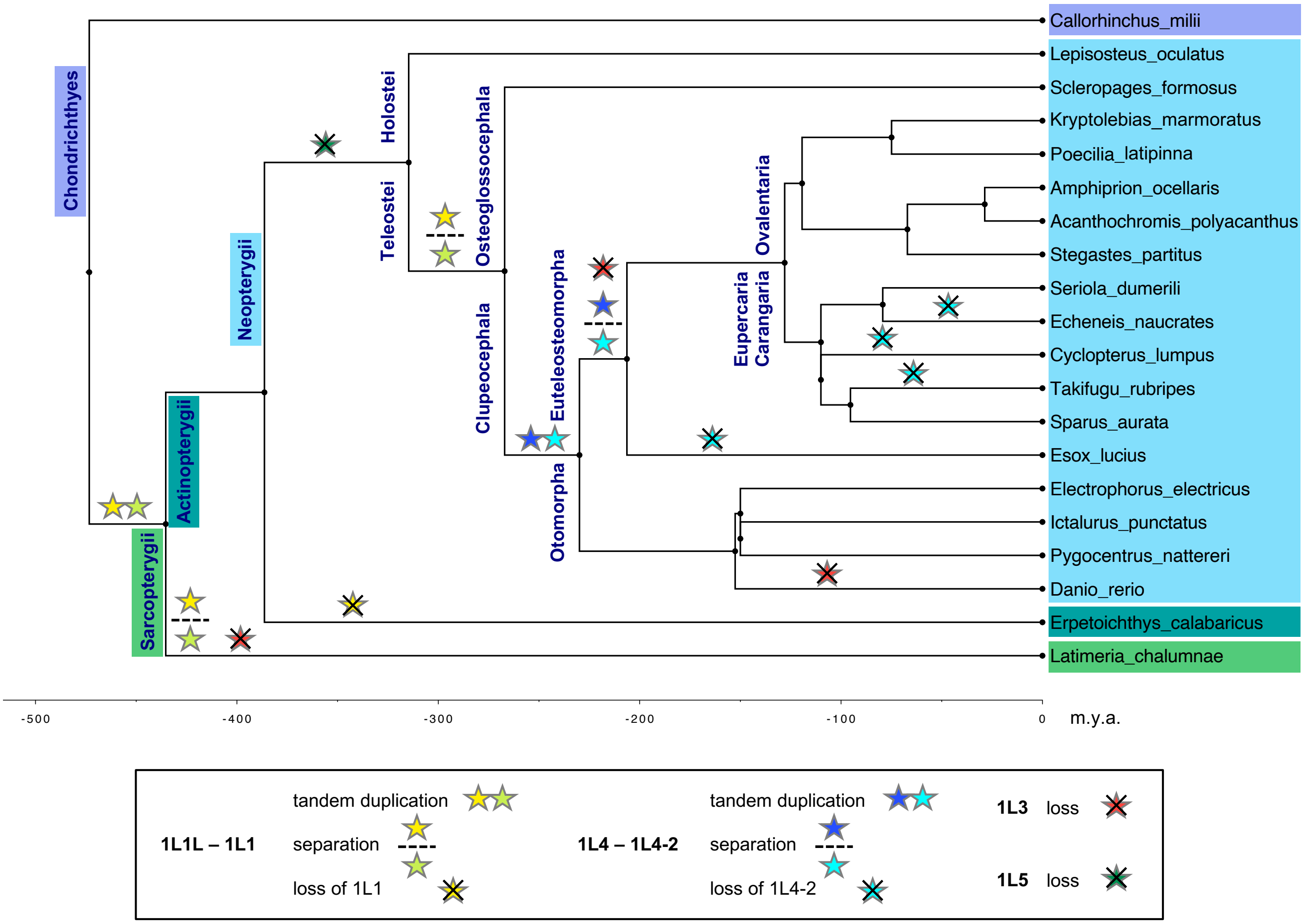

Figure S4. Chronogram of jawed fish phylogeny derived from TimeTree. Tandem duplications of DNase1L1L (1L1L – 1L1) and DNase1L4 (1L4 – 1L4-2) and successive separations, and loss of 1L1, 1L4-2, 1L3, and 1L5 are mapped on the relevant branches. For the dating of species, *Aplocheilus panchax* and *Poecilia reticulata* were used instead of *Kryptolebias marmoratus* and *Poecilia latipinna*, respectively. Genome scale synteny analysis revealed that *DNase1L4* and *DNase1L4-2* are located closely in tandem, arranged in opposite orientation (tail-to-tail) in Otomorpha, while they are separated in Euteleosteomorpha. Within the latter group, *DNase1L4-2* was generally maintained in Ovalentaria, but was frequently lost in Carangaria (e.g. *Echeneis naucrates*) and Eupercaria (e.g. *Takifugu rubripes* and *Cyclopterus lumpus*).

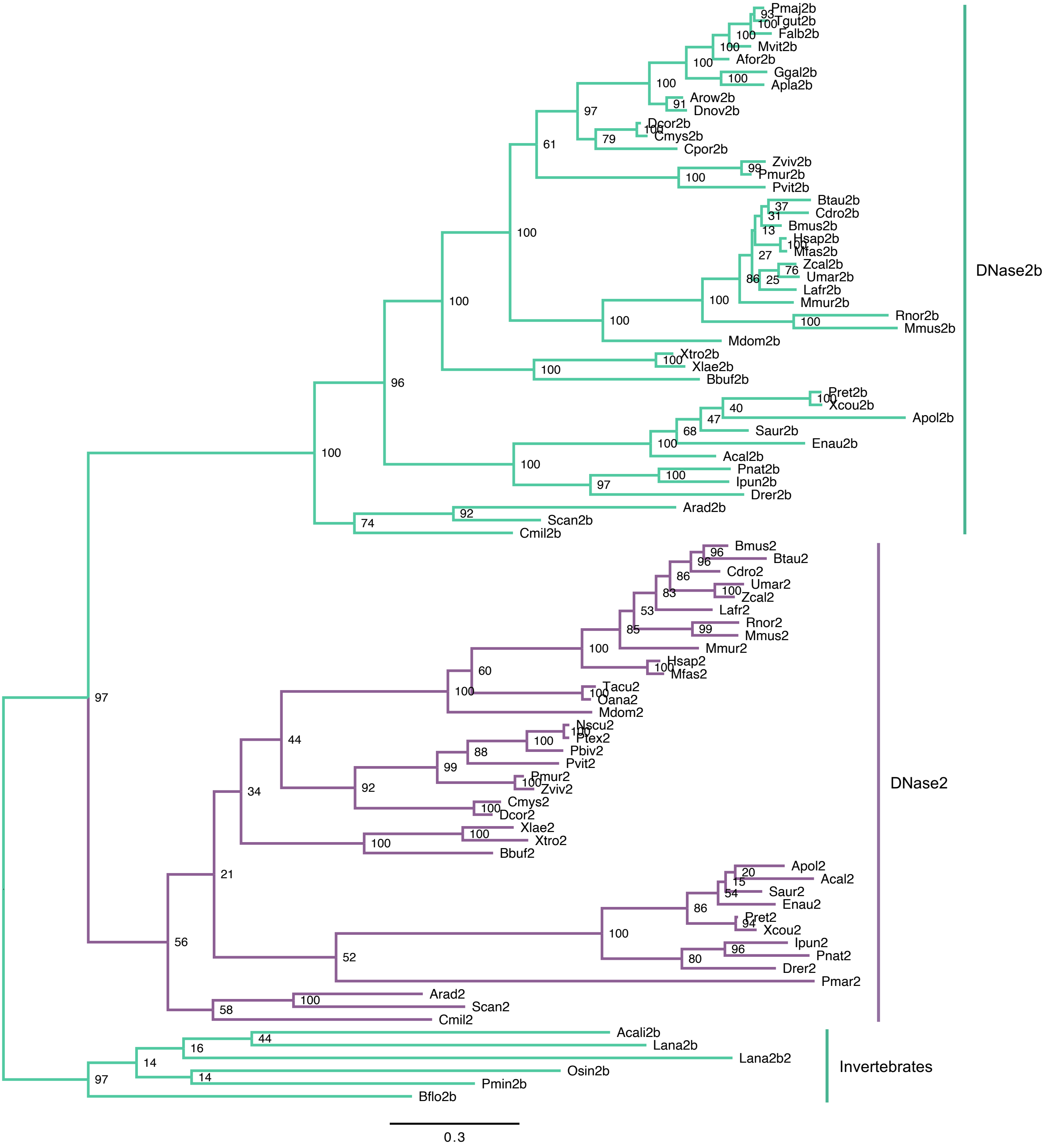

Figure S5. Maximum-likelihood phylogeny of 86 DNase2 family proteins inferred with the LG substitution model. Sequences are labeled with the abbreviated taxon name followed by the DNase2 family member identifier according to our classification. Invertebrate sequences were used to root the tree. Bootstrap values are shown at the internal nodes. Scale bar, substitution/site.

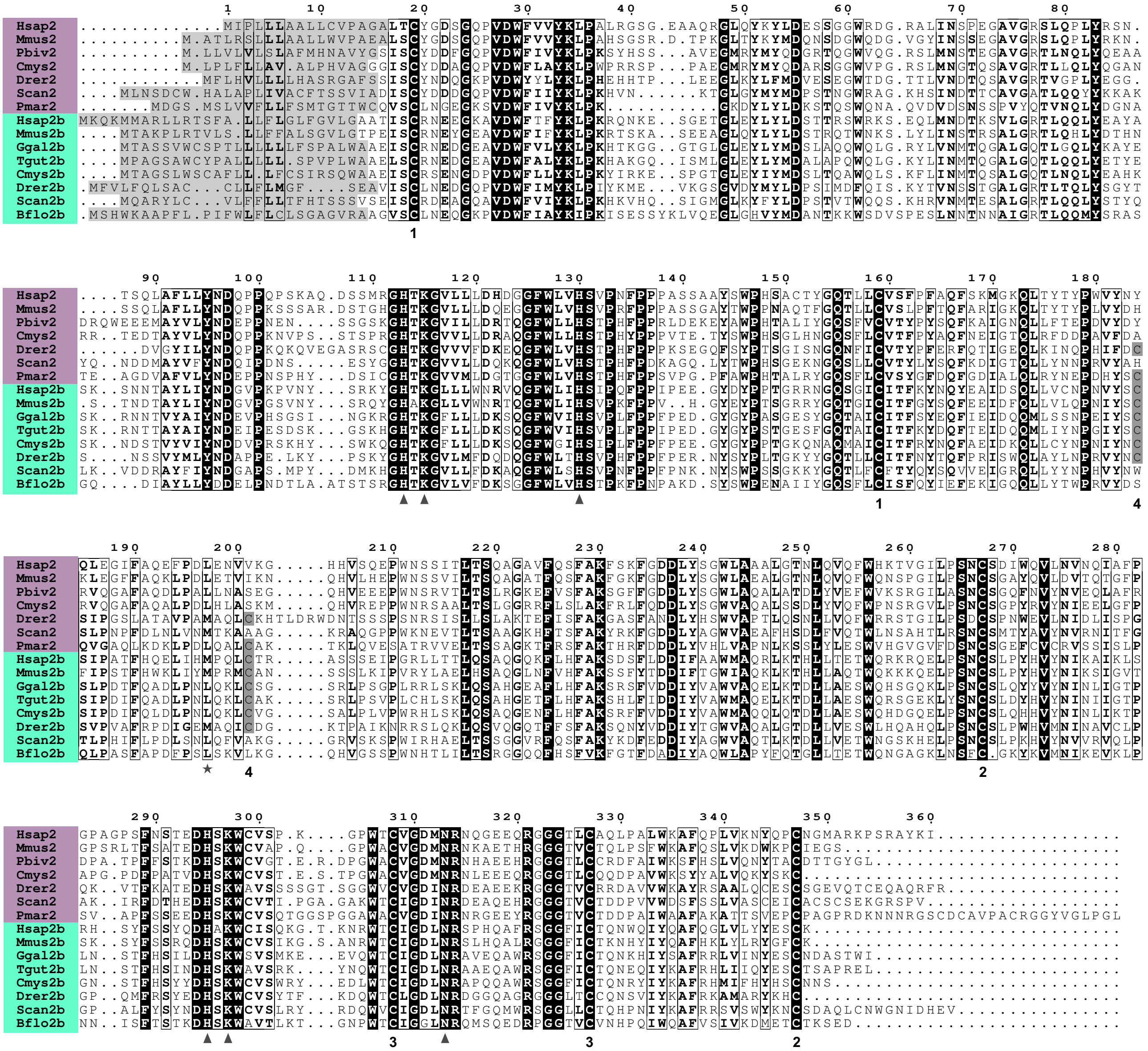

Figure S6. Multiple alignment of DNase2 family sequences from selected species. The catalytic residues of the PLD domains are indicated with triangles; the conserved cysteines pairs forming disulfide bonds are indicated with the same number, and the cysteines pair conserved only in DNase2b is also highlighted in dark gray; the signal peptides predicted with SignalP 5.0 are highlighted in light gray; the first methionine of DNase2b-S is indicated with a star (see Fig.6).

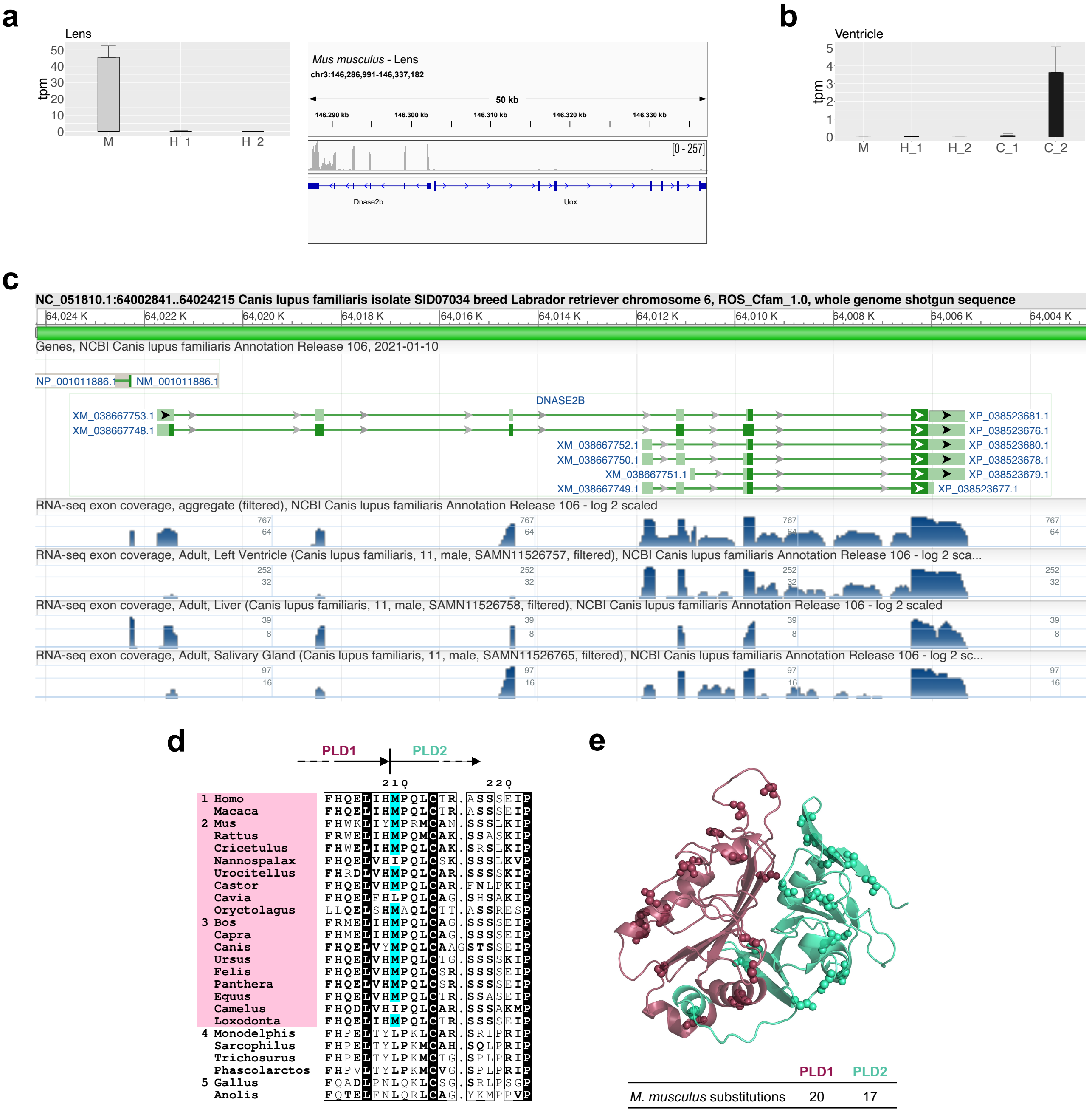

Figure S7. Expression and conservation of DNase2b isoforms. (**a**) Left panel: Gene expression levels of *DNase2b* in the lens of *M. musculus* (M) and *H. sapiens* (H) derived from RNA-seq data analysis. Right panel: Reads mapping to *DNase2b* showing expression of the long isoform in the mouse lens. (**b**) NCBI sequence-viewer representation of the genomic region on *Canis lupus familiaris* chromosome 6 encompassing *DNase2b* gene. Gene exon structure is represented by green segments. Blue bars represent RNA-seq exon coverage (log2 scaled) for aggregate and specific tissue datasets, showing expression of the short isoform of *DNase2b* in the ventricle, and expression of the long isoform in the liver, and salivary glands. (**c**) Gene expression levels of *DNase2b* in the ventricle of *M. musculus* (M), *H. sapiens* (H), and *C. lupus familiaris* (C) derived from RNA-seq data analysis. The suffix 1 and 2 refer to the long and short isoforms, respectively. (**d**) Portion of a multiple alignment of DNase2b proteins from vertebrate species showing conservation of the first methionine (cyan highlight) of DNase2b-S in placental mammals (pink highlight) with a few exceptions. The end of the PLD1 domain and the start of the PLD2 domain are shown above the alignment. The sequences are grouped according to the conservation pattern observed in the alignment: 1, Primates; 2, Rodents; 3 other Placentals; 4, Marsupials; 5, Sauropsids. (**e**) 3D model of *M. musculus* DNase2b structure the AlphaFold database (AF-Q9QY48-F1). Mutations at conserved positions in DNase2b of placental mammals are shown in spheres.

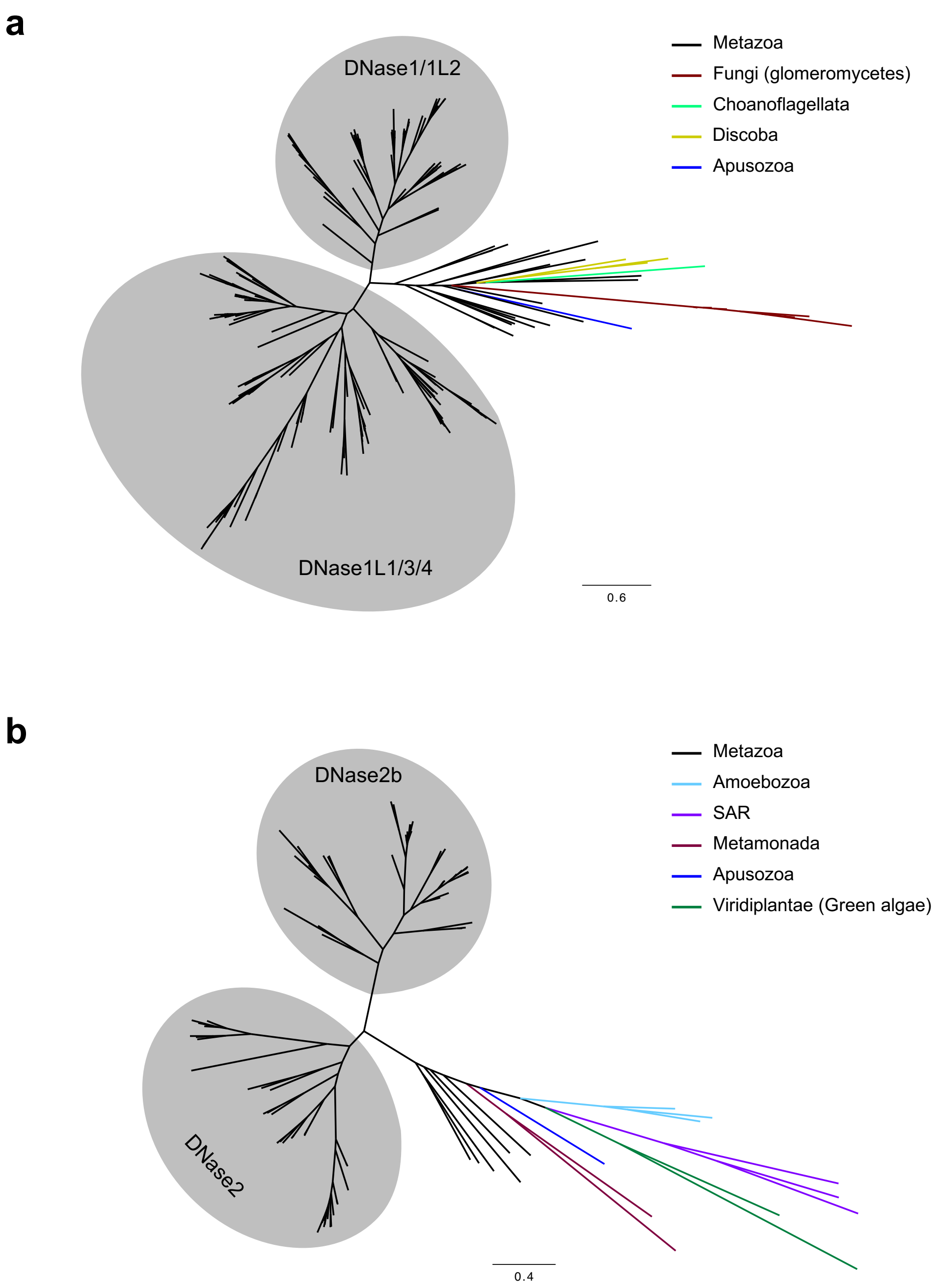

Figure S8. Phylogeny of DNases in eukaryotes. Unrooted maximum-likelihood phylogeny of (**a**) 212 DNase1 family proteins (excluding DNase1L5 group and amphibian sequences) and (**b**) 97 DNase2 family proteins.

Table S1. Expression profile of DNase1 and DNase2 from two human RNA-seq datasets from NCBI. Gene expression levels are given as RPKM (Reads per Kilobase per Million mapped reads).

	DNase1		DNase1L1		DNase1L2		DNase1L3		DNase2		DNase2b	
**Sample**	**A**	**B**	**A**	**B**	**A**	**B**	**A**	**B**	**A**	**B**	**A**	**B**
Adrenal gland	0.62	2	1	4	0.04	0.23	5	15	0	9	0.03	0.02
Brain	0.82	2	0.54	2	0.27	0.6	0.05	0.04	1	8	0.01	0.04
Heart	0.8	2	2.82	7	0.02	0.04	0.33	4	1	7	0	0.01
Kidney	2	5	2	5	0.06	0.42	2	14	2	7.77	0	0.03
Liver	0.67	1	0.42	2	0.01	0.08	32.14	62	3	9	0.01	0.09
Lung	0.58	2	2	6	0.01	0.16	1	9	5	17	0.36	0.45
Pancreas	n.a.	3	n.a.	0.65	n.a.	0.1	n.a.	0.11	n.a.	4	n.a.	0.04
Placenta	0.54	2	0.87	5	0.11	0.44	0.69	3	1	10	0	0.02
Prostate	1.22	3	1	4	0.08	0.35	0.31	0.81	3	13	0.4	1
Salivary gland	0.44	1	0.6	2	0.09	0.13	0.16	0.39	2	6.24	5	19
Skeletal muscle	0.94	n.a.	5	n.a.	0.13	n.a.	0.07	n.a.	1	n.a.	0.04	n.a.
Skin	n.a.	2	n.a.	2	n.a.	3	n.a.	2	n.a.	8.68	n.a.	0
Small intestine	4	11.6	0.92	4	0.03	0.15	5	20	2	6	0.01	0.03
Spleen	0.54	2	0.91	7	0.05	0.36	26	118	1	10	0.02	0.05
Stomach	0.81	2	0.75	4	0.08	0.22	1	2	2	11	0.02	0.03
Testis	n.a.	3	n.a.	2	n.a.	0.28	n.a.	2.39	n.a.	4	n.a.	0.11
Thymus	2	n.a.	0.62	n.a.	0.31	n.a.	3.56	n.a.	2	n.a.	0	n.a.
Thyroid	0.74	2	1	6.61	0.16	0.47	1	5	4	23	0.43	0.03
Trachea	0.38	n.a.	0.57	n.a.	0.03	n.a.	0.47	n.a.	2	n.a.	0.05	n.a.
Uterus	0.64	n.a.	0.69	n.a.	0.02	n.a.	0.19	n.a.	2	n.a.	0	n.a.

A: RNA-seq of total RNA from 20 human tissues

B: HPA RNA-seq normal tissues

Gene expression levels are given as RPKM (Reads per Kilobase per Million mapped reads)

Table S2. Sequence read archive IDs of the RNA-seq data analyzed for DNase gene expression.

**Skin**	*Gallus gallus*	SRR1265950_1.fastq.gz, SRR1265950_2.fastq.gz
		SRR1265951_1.fastq.gz, SRR1265951_2.fastq.gz
		SRR1265952_1.fastq.gz, SRR1265952_2.fastq.gz
	Homo sapiens	SRR15355253_1.fastq.gz, SRR15355253_2.fastq.gz
		SRR15355255_1.fastq.gz, SRR15355255_2.fastq.gz
		SRR15355257_1.fastq.gz, SRR15355257_2.fastq.gz
**Kidney**	*Gallus gallus*	ERR5101051_1.fastq.gz, ERR5101051_2.fastq.gz
		ERR5101045_1.fastq.gz, ERR5101045_2.fastq.gz
		ERR5101054_1.fastq.gz, ERR5101054_2.fastq.gz
	Homo sapiens	SRR8209856_1.fastq.gz, SRR8209856_2.fastq.gz
		SRR8209858_1.fastq.gz, SRR8209858_2.fastq.gz
		SRR8209860_1.fastq.gz, SRR8209860_2.fastq.gz
**Lens**	*Mus musculus*	SRR15328933_1.fastq.gz, SRR15328933_2.fastq.gz
		SRR15328934_1.fastq.gz, SRR15328934_2.fastq.gz
		SRR15328935_1.fastq.gz, SRR15328935_1.fastq.gz
	Homo sapiens	SRR12136461_1.fastq.gz, SRR12136461_2.fastq.gz
		SRR12136460_1.fastq.gz, SRR12136460_2.fastq.gz
		SRR12136459_1.fastq.gz, SRR12136459_2.fastq.gz
**Liver**	*Mus musculus*	SRR13571111_1.fastq.gz, SRR13571111_2.fastq.gz
		SRR13571112_1.fastq.gz, SRR13571112_2.fastq.gz
		SRR13571113_1.fastq.gz, SRR13571113_2.fastq.gz
	Homo sapiens	SRR15661720_1.fastq.gz, SRR15661720_2.fastq.gz
		SRR15661721_1.fastq.gz, SRR15661721_2.fastq.gz
		SRR15661722_1.fastq.gz, SRR15661722_2.fastq.gz
**Salivary Glands**	*Mus musculus*	SRR12415652_1.fastq.gz, SRR12415652_2.fastq.gz
		SRR12415653_1.fastq.gz, SRR12415653_1.fastq.gz
		SRR12415654_1.fastq.gz, SRR12415654_1.fastq.gz
	Homo sapiens	SRR10898042_1.fastq.gz, SRR10898042_2.fastq.gz
		SRR10898043_1.fastq.gz, SRR10898043_2.fastq.gz
		SRR10898045_1.fastq.gz, SRR10898045_2.fastq.gz
**Alveolar Macrophages**	*Mus musculus*	ERR4436755_1.fastq.gz, ERR4436755_2.fastq.gz
		ERR4436756_1.fastq.gz, ERR4436756_2.fastq.gz
		ERR4436757_1.fastq.gz, ERR4436757_2.fastq.gz
	Homo sapiens	SRR13198616_1.fastq.gz, SRR13198616_2.fastq.gz
		SRR13198625_1.fastq.gz, SRR13198625_2.fastq.gz
		SRR13198650_1.fastq.gz, SRR13198650_2.fastq.gz
**Ventricle**	*Homo sapiens*	SRR13417599_1.fastq.gz, SRR13417599_2.fastq.gz
		SRR13417600_1.fastq.gz, SRR13417600_2.fastq.gz
		SRR13417601_1.fastq.gz, SRR13417601_2.fastq.gz
	Mus musculus	SRR13396357_1.fastq.gz, SRR13396357_2.fastq.gz
		SRR13396358_1.fastq.gz, SRR13396358_2.fastq.gz
		SRR13396359_1.fastq.gz, SRR13396359_2.fastq.gz
	Canis lupus familiaris	SRR5889336_1.fastq.gz, SRR5889336_2.fastq.gz
		SRR8997008_1.fastq.gz, SRR8997008_2.fastq.gz
		SRR8997037_1.fastq.gz, SRR8997037_2.fastq.gz
